# The direct and indirect effects of COVID‐19 pandemic in a real‐life hematological setting

**DOI:** 10.1002/cnr2.1358

**Published:** 2021-03-03

**Authors:** Maria Condom, Alberto Mussetti, Clara Maluquer, Rocío Parody, Eva González‐Barca, Montserrat Arnan, Adaia Albasanz‐Puig, Helena Pomares, Maria Queralt Salas, Itziar Carro, Marta Peña, Victòria Clapes, Cristina Baca Cano, Ana Carla Oliveira Ramos, Gabriela Sanz‐Linares, Gabriel Moreno‐González, Santiago Mercadal, Concepcion Boqué, Carlota Gudiol, Eva Domingo‐Domènech, Anna Sureda

**Affiliations:** ^1^ Clinical Hematology Department Institut Català d'Oncologia—Hospital Duran i Reynals Barcelona Spain; ^2^ Institut d'Investigació Biomèdica de Bellvitge (IDIBELL) Barcelona Spain; ^3^ Intensive Care Medicine Bellvitge University Hospital, IDIBELL, University of Barcelona Barcelona Spain; ^4^ Spanish Network for Research in Infectious Diseases (REIPI RD16/0016/0001) Instituto de Salud Carlos III Madrid Spain; ^5^ Infectious Diseases Department Bellvitge University Hospital, IDIBELL, University of Barcelona Barcelona Spain; ^6^ Universitat de Barcelona Barcelona Spain

**Keywords:** COVID‐19, hematology, leukemia, lymphoma, SARS‐CoV‐2, telemedicine

## Abstract

**Background:**

Clinical outcomes of novel coronavirus 2019 disease (COVID‐19) in onco‐hematological patients are unknown. When compared to non‐immunocompromised patients, onco‐hematological patients seem to have higher mortality rates.

**Aims:**

We describe the characteristics and outcomes of a consecutive cohort of 24 onco‐hematological patients with COVID‐19 during the first month of the pandemic. We also describe variations in healthcare resource utilization within our hematology department.

**Methods and Results:**

Data from patients between the first month of the pandemic were retrospectively collected. Clinical and logistic data were also collected and compared with the average values from the prior 3 months of activity. Prevalence of COVID‐19 in our hematological population was 0.4%. Baseline characteristics were as follows: male sex: 83%, lymphoid diseases: 46%, median age: 69 (22‐82) years. Median follow‐up in survivors was 14 (9‐28) days and inpatient mortality rate was 46%. Average time to moderate/severe respiratory insufficiency and death were 3 (1‐10) and 10 (3‐18) days, respectively. Only 1 out of every 12 patients who developed moderate to severe respiratory insufficiency recovered. Upon univariate analysis, the following factors were associated with higher mortality: age ≥ 70 years (*P* = .01) and D‐dimer ≥900 mcg/L (*P* = .04). With respect to indirect effects during the COVID‐19 pandemic, and when compared with the prior 3 months of activity, inpatient mortality (excluding patients with COVID‐19 included in the study) increased by 56%. This was associated with a more frequent use of vasoactive drugs (+300%) and advanced respiratory support (+133%) in the hematology ward. In the outpatient setting, there was a reduction in initial visits (−55%) and chemotherapy sessions (−19%). A significant increase in phone visits was reported (+581%).

**Conclusion:**

COVID‐19 pandemic is associated with elevated mortality in hematological patients. Negative indirect effects are also evident within this setting.

## 1 INTRODUCTION

The novel coronavirus SARS‐CoV2 recently emerged as a global threat.^1^ High hospitalization (around 20% of diagnosed cases) and mortality rates (2%‐3%) of patients with COVID‐19 have led to an unprecedented burden on healthcare systems.^2^, ^3^ Indeed, the impact of COVID‐19 has even prevented healthcare professionals from continuing normal utilization practices of healthcare resources for all other health matters unrelated to the pandemic. Evidence of such can be seen in emergency departments and other vital services such as onco‐hematology departments.^4^, ^5^ Recent reports from Chinese colleagues showed how symptomatic COVID‐19 is perhaps associated with higher mortality in the oncological population, with a range of 5% to 35%.^6^, ^7^, ^8^ In our study, we describe how this pandemic directly affects our hematological patients. Furthermore, we analyze the indirect effects of such pandemic on our hematology department in terms of inpatient and outpatient activity of a population without COVID‐19 infection. With these data, we aim to further our understanding of the direct and indirect impacts of COVID‐19 on onco‐hematological patients and departments to facilitate the conception of effective contingency plans for future viral outbreaks.

## 2 PATIENTS AND METHODS

### 2.1 Data sources and statistics

The Institut Català d'Oncologia—Hospital Duran i Reynals (Barcelona, Spain) is a tertiary oncology referral center for adult patients, with a hospitalization ward of 26 beds dedicated to hematologic patients, attending to a population of almost 1.5 million people. In the first part of the study, we analyzed the direct effects of COVID‐19 on a consecutive series of onco‐hematological patients. We retrospectively collected clinical data from patients between March 13, 2020 (the first day of government‐imposed restrictions related to COVID‐19 in Spain) and April 12, 2020. Data cut‐off date was April 19, 2020. Data were collected via a retrospective chart review. All patients were ≥18 years old and had an onco‐hematological malignancy. COVID‐19 diagnosis and response were confirmed in accordance with World Health Organization criteria.^9^ Hematological disease type and status were defined per international standard criteria.^10^, ^11^ In the second part of the study, we described the indirect effects of COVID‐19 on inpatient and outpatient activity in our department. Clinical and logistic data from both types of activity were compared with average values calculated from the prior 3 months (between December 13, 2019, and March 12, 2020).

The primary endpoint of the study was to describe the all‐cause in‐hospital mortality rate in patients with hematologic neoplasms and a confirmed diagnosis of COVID19 infection, defined as the proportion of patients with COVID‐19 who died to the overall cohort. Secondary endpoints included: description of patient characteristics, average time from diagnosis to moderate/severe respiratory insufficiency (defined as requirement of oxygen support other than nasal cannula, 3 L/m or pO_2_/FiO_2_ < 300 mmHg), and average time from diagnosis to death. Descriptive data were reported as counts and percentages. The proportion of deaths was compared among baseline characteristics using chi‐squared test or Fisher's exact tests depending on the number of events. The following variables were considered: female vs male, age ≥ 70 years, ongoing chemotherapy (defined as last chemotherapy within the last 30 days) vs follow‐up, active disease vs cured disease, 0‐1 line of therapy vs ≥1, presence of comorbidities (mostly cardiovascular risk factors and chronic heart, lung, and kidney disease) vs no comorbidities, D‐dimer ≥900 mcg/L (median of study population) vs <900 mcg/L, radiological evidence of low tract respiratory infection in chest X‐ray vs negative radiological findings. The analysis was performed with STATA statistical software version 13.0.^12^

For patients without COVID‐19, a description of healthcare resource utilization between the first month of the COVID‐19 pandemic and the average of the prior 3 months was provided. For the inpatient setting, the following variables were analyzed: total number of patients admitted, non‐COVID‐19 inpatient mortality rate, median days of hospitalization, and the number of hematopoietic cell transplants (HCT) performed (autologous and allogeneic). Also, we described the number of patients without COVID‐19 infection who underwent sub‐intensive care procedures in the hematology ward (vasoactive drugs and high‐flow nasal cannula), as well as the number of patients who required intensive care unit (ICU) admission. For the outpatient setting, the analysis was divided into the following clinical units: HCT, acute leukemia and myelodysplastic syndromes, myeloproliferative diseases, lymphoid malignancies, multiple myeloma and other monoclonal gammopathies, and interventional clinical trials. We reported the following for each unit: the number of total visits, initial visits, in‐person follow‐up visits, phone visits, and daily sessions of chemotherapy/supportive therapy administered in the outpatient clinic.

### 2.2 Structural reorganization due to COVID‐19

Our institution is a tertiary oncology referral hospital for adult patients, comprising four clinical departments (Medical Hematology, Medical Oncology, Palliative Care, and Radiotherapy) and two services (Radiology and Pharmacy). Each department has its own ward and outpatient visitor area; however, outpatient therapy space is shared. Furthermore, the hospital has its own infectious disease specialists, and endocrinology and nutrition staff. For all other services, including the intensive care unit, the hospital collaborates with Hospital Universitari de Bellvitge (Barcelona, Spain). Due to the COVID‐19 pandemic, our center and activities underwent structural reorganization per regional, national, and international recommendations.^13^, ^14^ At the outpatient level, reduction of patient volume was sought with the aim to minimize as best as possible the number of outpatient chemotherapies during the first 2 weeks of the pandemic. First, chemotherapy sessions inside and outside the context of clinical trials were initiated only if patients were considered as high risk for progression. For myeloid malignancies and acute myeloid leukemia (AML), induction chemotherapies were continued and consolidation cycles were performed in an inpatient setting to reduce the number of hospital trips for the patient. For lymphoid malignancies, only severely symptomatic patients or those at high risk of developing complications started chemotherapy. Also, treatments were switched to an oral presentation whenever possible (eg, intramuscular methotrexate was changed to oral methotrexate for acute lymphoblastic leukemia maintenance) and nonessential treatments were postponed (eg, bisphosphonate therapy and intravenous iron therapy). For transplant and cell therapy, the following categories were considered high risk: allogeneic HCT for acute myeloid leukemia, aplastic anemia, any advanced stage of lymphoma not in complete remission, no further option of another cycle of chemotherapy, patients included in clinical trials, and chimeric antigen receptor cell candidates. New infusion dates were proposed in order to have no more than one allogeneic HCT per week. Cryopreservation of allogeneic products from related and non‐related donors was done prior to patient admission and conditioning in accordance with local (Registro de Donantes de Médula Ósea) and international (European Bone Marrow Transplantation Society) recommendations.^13^, ^14^, ^15^ Nonessential visits were substituted with phone visits whenever possible.

Palliative care and the emergency units of the hospital were converted into COVID‐19 wards. In order to prevent nosocomial COVID19 outbreaks in a high‐risk ward, nasopharyngeal swabs for COVID19 were performed in all patients 24 to 48 hours before their scheduled admission. Due to the restrictive triage protocol, no change in room pressure was implemented. No family members were authorized to stay with patients during their hospital stay. Additionally, physicians were separated into two different teams for each ward service (HCT, myeloid disease, and lymphoid disease) to minimize the loss of workforce due to a possible contagion. Outpatient staff was physically confined to specific areas. Teleworking was encouraged for all activities not requiring physical presence at the hospital. Prior agreement with the ICU to have a referral intensive care specialist in the hematology ward was made. This strategy was implemented with the objective to delay transfers of critical patients and assume more clinically complex cases, defined as those patients who require vasoactive drug support and/or present with rapid acute respiratory insufficiency and need noninvasive ventilation with high‐flow oxygen therapy. Patients admitted to the onco‐hematological ward had the same pharmacological treatments available as those admitted to the ICU, including tocilizumab and high‐dose corticosteroids.

## 3 RESULTS

### 3.1 Cohort description of patients with COVID‐19

Taking into consideration patients followed‐up in the last 2 years in our hematology department (n = 6779), the expected prevalence of COVID‐19 in our oncological population was 0.4%. This figure is similar to that reported in the general population of the same geographic area (0.5%).^16^ Of the 25 patients identified, one was excluded from survival analysis due to a non‐oncological disease (sickle cell disease). Upon analysis, only one patient was still hospitalized. Details concerning patient characteristics and baseline conditions, COVID‐19 disease presentation, treatment, and outcomes can be found in Table 1. Median age of patients was 69 years (22‐82); the majority of patients were male (83%); and lymphoid malignancies were the most common hematological disease (46%). Only two patients were neutropenic upon diagnosis. Median follow‐up in survivors was 14 days (7‐29). Mortality rate was 46%, higher than in the general population (10%).^16^ Eight of 24 patients (33%) required advanced respiratory support (more than nasal cannula 3 L/min). Average time from diagnosis to moderate to severe respiratory insufficiency was 3 days (1‐10). Average time from diagnosis to death was 10 days (3‐18). Upon univariate analysis, the only factors with a significant effect on these outcomes were age ≥ 70 years (*P* = .01), D‐dimer ≥900 mcg/L (*P* = .04), and female sex (*P* = .02). Of the 17 potential patients who developed respiratory insufficiency, eight required advanced respiratory support. One patient required endotracheal intubation and ICU admission; two received non‐invasive mechanical ventilation in the hematology ward and emergency department, respectively. Five patients received high flux nasal cannula in the ward.

**TABLE 1:** Clinical characteristics of patients and disease outcomes

Baseline characteristics		N. of patients (n = 24)
Median age, years (range)		69 years (22–82)
Male sex, n (%)		20/24 (83%)
Presence of comorbidities, n (%)		17/24 (71%)
Type of malignancy, n (%)		
	Lymphoid	11/24 (46%)
	CLL	3/11
	DLBCL	2/11
	MCL	1/11
	HL	1/11
	FL	1/11
	LGL leukemia	1/11
	tMZL	1/11
	AITL	1/11
	Plasma cell dyscrasia	6/24 (25%)
	MM	5/6
	SA	1/6
	Myeloid	7/24 (29%)
	AML	5/7
	MDS	1/7
	CMML	1/7
Number of lines of treatment received, n (%)		
	0–1	15/24 (62.5%)
	>1	9/24 (37.5%)
Active disease, n (%)		20/24 (83%)
On active treatment, n (%)		14/24 (58%)
COVID‐19 characteristics upon admission		
Fever, n (%)		21/24 (86%)
Oxygen support requirement, n (%)		2/24 (8%)
Laboratory findings, n (%)		
	Lymphopenia <1000 × 10^9^/L	14/24 (58%)
	Neutropenia <1000 × 10^9^/L	2/24 (8%)
	D‐dimer >250 μg/L	20/24 (83%)
	LDH > 220 (U/L)	15/22 (68%) 2 missing cases
	CPR >10 mg/L	19/23 (83%) 1 missing case
Radiological findings in chest radiograph, n (%)		
	Normal	10/24 (42%)
	Lobar infiltrate/consolidation	2/24 (8%)
	Bilateral interstitial infiltrates/consolidations	12/24 (50%)
Treatment		
COVID‐19‐targeted therapy, n (%)		
	None	2/24 (8%)
	HCQ only	8/24 (33%)
	HCQ + L/R	13/24 (54%)
	HCQ + D/R	1/24 (4%)
Inflammatory syndrome‐targeted therapy, n (%)		
	None	17/24 (71%)
	Tocilizumab only	4/24 (17%)
	Tocilizumab + steroids	1/24 (4%)
	Interferon B	2/24 (8%)
Maximum respiratory support, n (%), %^a^		
	None	9/24 (37.5%)
	Nasal cannula	3/24 (12.5%)
	Venturi mask	2/24 (8%)
	Monaghan reservoir	2/24 (8%)
	HFNC	5/24 (21%)
	NIMV	2/24 (8%)
	EI	1/24 (4%)
Complications during hospitalization		
Acute respiratory distress syndrome, n (%)		12/24 (50%)
Other documented concurrent infections, n (%)		
	Bacterial respiratory tract	3/24^b^ (12%)
	Bacterial, non‐respiratory tract	2/24^c^ (8%)
	Non‐bacterial infections	0/24 (0%)
Acute kidney injury AKIN III		2/24 (8%)
Stroke		1/24 (4%)
Skin rash		3/24 (12.5%)
Outcomes		
Clinical outcomes, n (%)		
	Cured and discharged	12/24 (50%)
	Death	11/24 (46%)
	Still admitted	1/24 (2%)
Median time from diagnosis to respiratory deterioration (days, range)		3 (1–10) days
Median time from diagnosis to death (days, range)		10 (3–18) days
Median follow‐up in survivors (days, range)		14 (9–28) days

Abbreviations: AITL, angioimmunoblastic T‐cell lymphoma; AKIN, Acute Kidney Injury Network; AML, acute myeloid leukemia; ASCT, autologous stem cell transplant; CLL, chronic lymphocytic leukemia; CMML, chronic myelomonocytic leukemia; CRP, C reactive protein; D/R, Darunavir/Ritonavir; DLBCL, diffuse large B‐cell lymphoma; EI, endotracheal intubation; FL, follicular lymphoma; HCQ, hydroxychloroquine; HCT, hematopoietic cell transplantation; HFNC, high flow nasal cannula; HL, Hodgkin lymphoma; LDH, lactate dehydrogenase; LGL, large granular lymphocytes; L/R, Lopinavir/Ritonavir; MCL, mantle cell lymphoma; MDS, myelodysplastic syndrome; MM, multiple myeloma; NIMV, noninvasive mechanical ventilation; SA, systemic amyloidosis; tMZL, transformed marginal zone lymphoma.

^a^ Nasal cannula administers up to 3 L oxygen/min; Venturi mask: 3‐15 L oxygen/min, at 25%‐50% FiO_2_; Monaghan reservoir: 15 L oxygen/min, 100% FiO_2_.

^b^ Bacterial respiratory tract superinfections: 2 *Haemophilus influenzae*, 1 *Branhamella catarrhalis*.

^c^ Bacterial, non‐respiratory tract infections: 1 *Clostridium difficile* colitis, 1 polymicrobial vulvar abscess (*Klebsiella pneumoniae*, *Staphylococcus aureus*).

### 3.2 Indirect effects of COVID‐19 on hematological outpatient and inpatient activity

**FIGURE 1:**
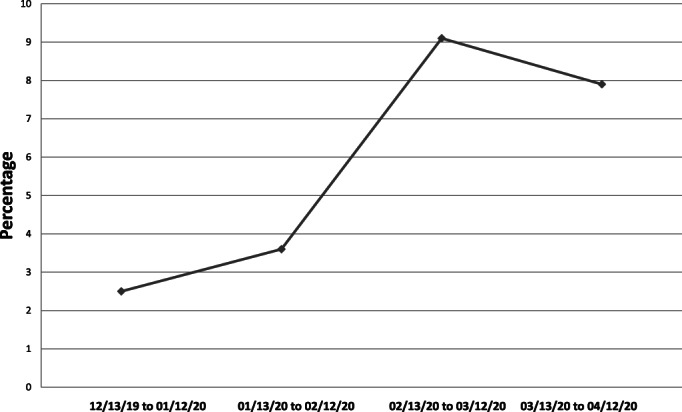
Inpatient mortality rate for patients without COVID‐19 infection during the first pandemic month and the prior 3 months

During the first month of pandemic restrictions, monthly inpatient activity of non‐COVID‐19 patients was affected (Table 2). The total number of admissions dropped by 35% and mortality rate of these patients increased by 56% (Figure 1). We observed an increased number of intensive care procedures performed in the hematology ward (use of vasoactive drugs +300%). Critically ill patients received on‐site sub‐intensive care. Finally, the number of autologous and allogeneic HCTs was reduced by 85% and 50%, respectively, when compared with the average calculated of the prior 3 months. A total of five HCTs were delayed (two autologous HCT for patients diagnosed with non‐Hodgkin lymphoma and systemic amyloidosis, and three allogeneic HCTs for patients diagnosed with AML, Hodgkin lymphoma, and multiple myeloma). Concomitantly, a total of three aphereses (two from unrelated donors and one from a related donor) was frozen during the study period.

**TABLE 2:** Differences in healthcare resources utilization between the prior 3 months and the COVID‐19 first month

	Average of prior 3 months (SD)	First COVID‐19 month	Variation (%)
Inpatient setting			
General			
Total admissions (n)	81 (±5.5)	53	−35
Total discharges (n)	80 (±3.0)	76	−5
Inpatient mortality (%)	5.0 (±3.5)	7.9	+56
Days of hospitalization (median)	9.9 (±1.13)	8.5	−14
Sub‐intensive/intensive care			
Patients using vasoactive drugs (n)	1 (±1.0)	4	+300
Patients using high‐flow nasal cannula (n)	3 (±2.6)	7	+133
Patient candidates for ICU admission (%)	4% (±3%)	9%	+130
Patients admitted to ICU on candidates (%)	100%	40%	−60
Mortality ICU (%)	40% (±35%)	100%	+157
HCT procedures			
ASCT (n)	7 (±3.0)	1	−85
AlloHCT (n)	4 (±1.7)	2	−50
Outpatient setting			
Global			
Total visits (n)	1395 (±195.3)	1405	+1
Number of first visits (n)	86 (±23.7)	39	−55
Number of in‐person follow‐up visits (n)	1097 (±204.5)	328	−70
Number of phone visits (n)	152 (±46.8)	1038	+581
Chemotherapy sessions and supportive therapy^a^ (n)	988 (±115.1)	803	−19
HCT unit			
Total visits (n)	129 (±24.5)	149	+16
Number of in‐person first visits (n)	14 (±4.6)	8	−43
Number of in‐person follow‐up visits (n)	102 (±12.5)	32	−69
Number of phone visits (n)	13 (±7.6)	109	+761
Acute leukemias and myelodysplasias			
Total visits (n)	200 (±66.0)	258	+29
Number of first visits (n)	11 (±4.6)	3	−73
Number of in‐person follow‐up visits (n)	155 (±63.2)	42	−73
Number of phone visits (n)	34 (±10.4)	213	+526
Chemotherapy sessions and supportive therapy^a^ (n)	601 (±51.6)	496	−17
Myeloproliferative diseases			
Total visits (n)	74 (±37.4)	86	+15
Number of first visits (n)	6 (±4.1)	1	−84
Number of in‐person follow‐up visits (n)	68 (±37.6)	19	−72
Number of phone visits (n)	9 (±3.8)	66	+607
Chemotherapy sessions and supportive therapy^a^ (n)	0	0	—
Lymphoid malignancies^b^			
Total visits (n)	324 (±23.0)	322	−1
Number of in‐person first visits (n)	29 (±3.2)	16	−44
Number of in‐person follow‐up visits (n)	278 (±25.1)	61	−78
Number of phone visits (n)	18 (±14.0)	245	+1261
Chemotherapy sessions and supportive therapy^a^ (n)	164 (±29.6)	147	−11
Multiple myeloma			
Total visits (n)	281 (±33.7)	230	−18
Number of first visits (n)	13 (±0.5)	3	−76
Number of in‐person follow‐up visits (n)	249 (±39.0)	11	−96
Number of phone visits (n)	19 (±5.8)	216	+1017
Chemotherapy sessions and supportive therapy^a^ (n)	223 (±33.8)	153	−31
Interventional clinical trials unit			
Total visits (n)	377 (±22.8)	360	−5
Number of first visits (n)	13 (±6.6)	8	−40
Number of in‐person follow‐up visits (n)	246 (±26.9)	163	−34
Number of phone visits (n)	59 (±5.2)	189	+220
Number of enrolled patients (n)	11 (±5.8)	87	−25
Total number of BM aspirates/biopsies (n)	107 (±22)	86	−20

Abbreviations: alloHCT, allogeneic hematopoietic cell transplant; ASCT, autologous stem cell transplant; BM, bone marrow; CLL, chronic lymphocytic leukemia; HL, Hodgkin disease; ICU, Intensive Care Unit; LPL, lymphoplasmacytic lymphoma; NHL, non‐Hodgkin lymphoma; WD, Waldenström disease.

^a^ One chemotherapy session comprises a single day of administering chemo. (Azacitidine 1 cycle = 7 sessions; R‐CHOP 1 cycle = 1 session). Transfusion support was counted into this category only for acute leukemias and myelodysplastic syndromes only. Zolendronate infusion was counted into this category for the multiple myeloma group.

^b^ CLL, NHL, HD, WD, LPL.

**FIGURE 2:**
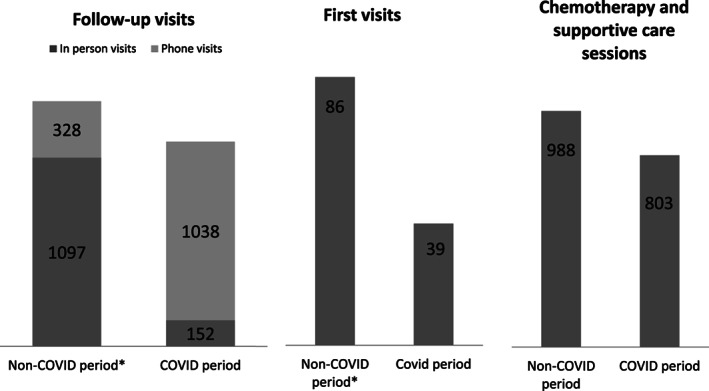
Outpatient activity between the COVID‐19 month and the average of the prior 3 months

Outpatient activity was severely affected during the COVID‐19 period. The total number of first visits dropped by 55% (Figure 2). In addition, the number of in‐person follow‐up visits decreased by 70%. Interestingly, the number of follow‐up phone visits increased by 581%. The average outpatient clinic activity (daily sessions of chemotherapy and supportive care) decreased by 19%. No changes were observed in relation to oral therapy administration. These changes were homogeneous in all subunits. In spite of 7 of 32 trials (with active enrollment) on hold during the first month of the COVID‐19 outbreak in Barcelona, the number of patients enrolled in clinical interventional studies did not decrease significantly. A reduction of 20% in bone marrow biopsies and marrow aspirates was reported. No COVID‐19 infections were registered among physicians.

## 4 DISCUSSION

We report for the first time a descriptive analysis of the direct and indirect effects of the COVID‐19 pandemic on a tertiary oncology referral center. Firstly, we performed an analysis of the characteristics of symptomatic COVID‐19 from a consecutive cohort of hematological patients. The prevalence of symptomatic COVID‐19 in our patients' population was comparable to that of the general population. This is different from what was previously reported in a Chinese study, which suggested a higher risk of contagion in the oncological population.^8^ The fact that patients diagnosed with hematological disorders already adopt preventive isolation strategies, such as social distancing, may have helped in keeping this prevalence stable. Most importantly, the mortality rate was higher than that in the general population of the same geographical area and time interval.^16^

Several factors could explain this difference. First of all, cancer is generally a disease of the elderly. Older age was associated with increased COVID‐19‐related mortality in almost all epidemiological studies. Considering that the median age of our study population was 69 years, this could have likely contributed to poor survival.^17^ Additionally, the presence of comorbidities is another well‐known risk factor.^18^ The majority of our patients had at least one chronic cardiorespiratory disease. Indeed, cancer also represents a severe comorbidity due to the disease itself, as well as the toxic effects of oncological treatments. Other reports described a higher COVID‐19‐related mortality in this population, ranging from 20% to 40%.^6^, ^7^, ^8^ ^19^ In a recent large study, hematological malignancy was associated with a higher risk of severe events in a COVID‐19 population.^19^

The use of different types of therapy could also influence this outcome. For example, B‐cell depletion by use of rituximab could potentially interfere with an adequate antibody response to the SARS‐CoV‐2. The use of checkpoint inhibitors capable of eliciting an increased immune response could have a role in favoring acute respiratory distress syndrome within this setting. More data are needed to clarify this hypothesis. In addition, being neutropenic could favor bacterial super‐infections during COVID‐19.

Nonetheless, such a high mortality rate appears superior to that of any other common seasonal respiratory virus (eg, influenza viruses, RSV), which usually does not exceed 10% to 20%, including in severely immunocompromised patients.^20^, ^21^ This may reflect the higher virulence of SARS‐CoV‐2 as observed in the general population.

We believe that the high mortality rate cannot be attributed exclusively to the COVID19, especially considering that 20/24 patients (83%) had an active underlying hematological neoplasm. However, in all patients who died, the leading cause of death was respiratory insufficiency, which makes us believe that COVID‐19 had a critical role on the poor patients' outcomes.

Our study also confirms elevated age and D‐dimer levels as risk factors associated with higher mortality.^17^ Female sex was not reported as a risk factor in other studies. However, these results were obtained from a small cohort of patients and should be confirmed in larger series. Apart from patient and virus‐related risk factors, it is possible that logistic factors could also play a role in COVID‐19 mortality. Even if this does not stand to be our case, patients requiring ICU admission were prioritized according to ethical regional or national guidelines. We can speculate that this prioritization might have disadvantaged onco‐hematological patients worldwide.

Finally, we suggest that onco‐hematological patients could deteriorate faster than the general population. This is true in terms of time from diagnosis to respiratory deterioration and death. A similar observation was reported elsewhere.^8^ Interestingly, we observed an inability to recover from moderate to severe respiratory insufficiency. In our series, 12 patients developed moderate to severe respiratory insufficiency. Of those, only one recovered. On the contrary, almost 80% of patients with COVID‐19‐related respiratory insufficiency in the general population will fully recover.^22^

Considering the indirect effects of COVID‐19, it is important to describe how such a pandemic was able to cause the collapse of a modern healthcare system. Following national and international recommendations, we reduced our daily activity and reserved it only for selected necessary cases. At an inpatient level, we observed an increased mortality in our patients who did not present with COVID‐19 infection. This could probably be explained by the need to attend to more palliative care patients in the hematology ward during the COVID‐19 period. To a lesser extent, a limited use of ICU and other essential hospital services could have played a role. Increased COVID‐19 mortality is in line with data from the national registry, which reported a significant increase in death from all causes during March 2020. As it concerns outpatient care, the number of first visits decreased significantly in all units (*see* Table 2). The drop was especially significant in the myeloid division. It is possible that this effect could be related to societal pressure to avoid hospital visits during this period and to fear that infection may be more probable in such an environment. Indeed, hospitals are considered high‐risk spots for contagion. We hypothesize that due to more unspecific symptoms, people with myeloid malignancies might have avoided an early medical consultation. Also, limited availability in radiology and surgical services could have contributed to significant delays in new diagnoses of lymphomas. Epidemiological studies will be needed to confirm this hypothesis and test another indirect effect of the COVID‐19 pandemic.

Our ability to effectively reduce the number of in‐person follow‐up visits was inversely related to a significant increase in phone visits in our outpatient clinics. Telemedicine has shown to be a feasible option within the onco‐hematological setting. Apart from lowering the probability of in‐hospital contagion during a pandemic, telemedicine should be implemented in the near future to minimize unnecessary hospital trips and thereby improve patients' quality of life. Administering chemotherapy and supportive care was mildly reduced. This indicates that chemotherapy is a medical procedure that can be delayed but in exceptional cases only. Cancer remains a lethal disease; oncological centers should guarantee the normal continuation of life‐saving procedures during an emergency situation.

In conclusion, COVID‐19 resulted in higher mortality in our cohort of hematological patients when compared to the general population of the same geographical region. Patients and disease factors could be involved in these poor outcomes; limited health resources during an emergency situation could also have a role. Enrollment in clinical trials should be encouraged in this setting.

Except for telemedicine and sub‐intensive care interventions in the ward, a significant reduction in almost all activities, including first visits, in a hematology department has been observed. The current indirect effects of the SAR‐CoV‐2 pandemic could potentially lead to additional negative effects on the hematological population (mostly in relation to decreased intensive care services for admitted patients and decreased diagnoses in the outpatient setting). Larger studies with a prolonged follow‐up will be necessary to understand in‐depth clinical characteristics of this new infection and its indirect effects. A more comprehensive view of how a pandemic could affect a hematological center in the modern era could facilitate the conception of improved contingency plans in the advent of health emergencies.

## CONFLICT OF INTEREST

The authors have declared no relevant conflicts of interest related to this study.

## AUTHOR CONTRIBUTIONS

All authors had full access to the data in the study and take responsibility for the integrity of the data and the accuracy of the data analysis. *Conceptualization*, M.C., A.M., C.M.; *Methodology*, A.M.; *Investigation*, M.C., A.M, C.M., R.P., E.G.B., M.A., A.A.P., H.P., M.Q.S., I.C., M.P., V.C., C.B., A.C.O., G.S.L., G.M.G., S.M., C.B., C.G., E.D.D.; *Formal Analysis*, M.C., A.M., C.M.; *Writing—Original Draft*, M.C., A.M., C.M., R.P., E.G.B., M.A., A.A.P., M.Q.S., G.M.G., E.D.D., A.S.; *Writing—Review & Editing*, M.C., A.M., C.M., A.S.; *Visualization*, M.C., A.S.; *Supervision*, A.S.; Project Administration: A.M.

## ETHICS STATEMENT

The Institutional Review Board (IRBs) of the Catalan Institute of Oncology‐Durán i Reynals Hospital approved this study and patients provided informed consent for data collection.

## Data Availability

The data that support the findings of this study are available on request from the corresponding author. The data are not publicly available due to privacy or ethical restrictions.
